# Increased risk of testis failure in testicular germ cell tumor survivors undergoing radiotherapy

**DOI:** 10.18632/oncotarget.23081

**Published:** 2017-12-07

**Authors:** Marco Ghezzi, Luca De Toni, Pierfrancesco Palego, Massimo Menegazzo, Elisa Faggian, Massimiliano Berretta, Francesco Fiorica, Maurizio De Rocco Ponce, Carlo Foresta, Andrea Garolla

**Affiliations:** ^1^ Unit of Andrology and Reproductive Medicine, Department of Medicine, University of Padova, Padova, Italy; ^2^ Department of Medical Oncology, National Cancer Institute, IRCCS Aviano, Aviano, Italy; ^3^ Department of Radiotherapy, University Hospital Sant'Anna, Ferrara, Italy

**Keywords:** sperm parameters, luteinizing hormone, vitamin D, parathormone, Leydig cells

## Abstract

Testicular germ cell tumors (TGCTs) are prevalent in males of reproductive age. Among the available therapeutic choices, pelvic radiotherapy (RT) and simple surveillance (SURV) are usually pursued. However, RT is considered to have life-threatening effects on testicular functions. In this study we sought to clarify this issue by evaluating sperm parameters and sex hormones in 131 TGCTs RT-treated-patients at both baseline (T0) and 12 (T1) and 24 months (T2) of follow-up. An age-matched group of 61 SURV patients served as control. Sperm parameters were comparable between SURV and RT at T0.

The RT group showed a significant reduction of all sperm parameters at T1 (all *P* values < 0.05 *vs* T0 and *vs* SURV at T1) and increased levels of sperm aneuploidies, with some degree of recovery at T2. On the other hand, despite normal levels of total testosterone being detected in both groups, luteinizing hormone (LH) levels in the RT group progressively increased at T1 and T2 with a relative risk of developing subclinical hypogonadism of 3.03 (95% CI: 1,50–6,11) compared to SURV. Again, compared to SURV, exposure to RT was associated with a 5.78 fold (95% CI: 2,91–11,48) risk of developing vitamin D insufficiency. These data suggest a likely RT-dependent impairment of the Leydig cell compartment.

## INTRODUCTION

Testicular germ cell tumors (TGCTs) are the most frequent cancer disease in young males aged from 15 to 40 years, with an outstanding increase in incidents over the past 50 years [1, 2]. The treatment of TGCTs has been a concrete success. Orchiectomy with postoperative radiotherapy (RT), platinum-based chemotherapy (CT) or just surveillance are associated with 5-year survival rates in excess of 95%. [3]. After successful cancer treatment, the perspective life expectancy of 40 to 50 years has accentuated the reduction of treatment-related toxicity and the maintenance of high therapeutic efficiency [4]. In seminoma patients, pelvic lymph node adjuvant-treatment with RT represents therapeutic choice depending on several clinical conditions such as tumor staging (particularly in low seminoma stage), prognostic factors and patient’s decision after oncological counseling, to cite a few [5, 6].

The risk/benefit ratio of RT has recently been queried because adverse effects of cancer treatment may develop during the treatment itself and persist during follow-up, from months to years after the completion of therapy [4]. This aspect is the most serious and potentially life threatening effect of cancer treatment. Particularly for RT, moderate radiation dose in infra-diaphragmatic stage I seminoma has been associated with radiation-induced malignancies located within or close to initial abdominal radiation fields (bladder, stomach, pancreas, and colon cancers). Nevertheless, the risks for solid cancer development after current cytotoxic treatment appears significantly reduced compared to “old-fashioned” RT, featured by larger radiotherapy fields and higher radiation doses than those used today [7, 8]. More recent causes of concern ascribed to RT are the negative side effects on testicular function, affecting both spermatogenesis and the global endocrine function of the testis, which may lead to oligo/azoospermia and hypogonadism. Indeed, whilst on a site, the germ cell compartment is known to be highly sensitive to chemotherapy [9], the Leydig cell compartment is also affected by radiation, resulting in subnormal/normal levels of serum testosterone values and/or increased luteinizing hormone (LH). This would be particularly relevant after orchiectomy in testicular cancer patients, in which the cytotoxic effect of ionizing radiation on the contralateral testis could prevent its compensatory growth. However, available studies report conflicting results to this regard and it is presently not possible to identify patients at high risk of long-term hypogonadism as a consequence of RT treatment [10].

The concept of male hypogonadism has recently been widened beyond the mere production of testosterone (T), since Leydig cells are involved in the release of other factors of endocrine value, such as 25-hydroxyvitamin D (25(OH) Vit D) with its known role in calcium-phosphorous metabolism [reviewed in 11]. In the present study we investigated the mid- and long-term development of clinical signs of testis derangements by evaluating testicular color Doppler ultrasound, sex hormones and calcium-phosphorous metabolism in a group of seminoma patients undergoing post-orchiectomy RT treatment and followed-up for the subsequent two years. In order to assess the impact of RT on sperm quality, we also investigated sperm aneuploidies. As controls, a group of seminoma patients who received time-matched surveillance only (SURV) was considered.

## RESULTS

### Clinical characteristics of the study group at baseline

One-hundred-ninety-two seminoma patients, 61 of whom received SURV and 131 RT, were retrospectively recruited. Clinical characteristics of patients at baseline are summarized in Table 1. The two groups of patients showed overlapping age at diagnosis (SURV 33.2 ± 5.9 years *vs* RT 32.9 ± 6.4 years; *P =* 0.774). The analysis of semen parameters showed no difference between the two groups in terms of sperm concentration, total sperm count, sperm motility and morphology. The analysis of the endocrine pattern, together with the evaluation of testis volume by color Doppler ultrasound and parameters, calcium-phosphorous metabolism also showed no significant difference at baseline between SURV and RT patients.

**Table 1:** Basal clinical characteristics of recruited TGCTs patients, undergoing surveillance only (SURV) or radio-therapy (RT) as post-orchiectomy treatment

	SURV (*N* = 61)	RT (*N* = 131)	*P* value
	Mean Value ( ± SD)	Mean Value ( ± SD)	
Age at diagnosis (yrs)	32.2 ± 5.9	32.9 ± 6.4	0.774
Sperm concentration (10^6^ cells/ml) [n.r. ≥39 × 10^6^ cells/ml]	42.5 ± 39.8	42.5 ± 43.9	0.496
Total sperm count (10^6^ cells) [n.r. ≥15 × 10^6^ cells]	111.8 ± 145.9	135.2 ± 158.4	0.156
Morphology (% of typical forms) [n.r. ≥4%]	13.8 ± 7.9	13.4 ± 5.6	0.895
Motility (% progressive motility) [n.r. ≥32%]	40.6 ± 19.3	38.9 ± 19.7	0.113
LH (IU/L) [n.r. 1–9 IU/L]	5.6 **±** 2.8	6.9 ± 3.6	0.063
FSH (IU/L) [n.r. 1–8 IU/L]	8.3 ± 4.6	7.1 **±** 3.8	0.174
Testosterone (nmol/L) [n.r. ≥10.4 nmol/L]	15.8 ± 4.8	16.1 ± 4.6	0.076
Testis volume (mL) [n.r.12–25 mL]	16.5 ± 6.3	19.3 ± 6.6	0.711
25OH Vit D (nmol/L) [nr. >50 nmol/L]	72.0 ± 32.5	68.7 ± 21.8	0.421
PTH (ng/L) [n.r. 17–73 ng/L]	38.9 ± 16.4	41.3 ± 21.1	0.089
1,25(OH)_2_ Vit D (nmol/L) [n.r. 43–148 pmol/mL]	93.8 ± 37.6	92.5 ± 31.2	0.524
Calcium (nmol/L) [n.r. 2.10–2.55 mmol/L]	2.37 ± 0.09	2.30 ± 0.11	0.612
Phosphorous (nmol/L) [n.r. 0.87–1.45 mmol/L]	1.09 ± 0.14	1.21 ± 0.18	0.094

Abbreviations: SD: standard deviation, n.r.: normal range, yrs: years, ml: milliliter, LH: luteinizing hormone, FSH: follicle stimulating hormone, IU: international units, L: liter, nmol: nanomole, 25OH Vit D: 25-hydroxy Vitamin D, PTH: parathormone, ng/dL: nanograms per deciliter, 1,25(OH)_2_ Vit D: 1,25-di-hydroxy Vitamin D.

### Analysis of semen parameters and sperm aneuploidies during the follow-up period

The time course of semen parameters of both SURV and RT during the two years of follow-up post-orchiectomy are reported in Table 2.

**Table 2:** Semen parameters during the follow-up period in patients undergoing surveillance only (SURV, *N* = 61) or radio-therapy (RT, *N* = 131) as post-orchiectomy treatment

	Sperm concentration (10^6^cells/mL)		Total sperm count (10^6^cells)		Progressive motility (%)		Normal sperm morphology (%)	
	SURV	RT	SURV	RT	SURV	RT	SURV	RT
T0	42.5 ± 39.8	42.5 ± 43.9	111.8 ± 145.9	135.2 ± 158.4	40.6 ± 19.3	41.9 ± 19.7	13.8 ± 7.9	13.4 ± 5.6
T1	47.1 ± 54.7	30.3 ± 42.7 ***a.d***	142.2 ± 122.4	88.3 ± 139.7 ***a.d***	43.8 ± 19.4	23.3 ± 19.3 ***b.c***	14.1 ± 5.8	10.6 ± 6.2 ***b.c***
T2	61.5 ± 47.1 ***c***	41.3 ± 46.2	181.3 ± 278.0 ***c***	116.8 ± 144.8	49.9 ± 21.7	28.6 ± 20.3 ***a.c***	16.4 ± 6.4 ***c***	12.9 ± 5.2 ***a***

Abbreviations: T0: baseline, T1: 1 year follow-up, T2: 2 years follow-up significance:

*a* = *P* < 0.05 *vs* corresponding time point of SURV.

*b* = *P* < 0.01 *vs* corresponding time point of SURV.

*c* = *P* < 0.05 *vs* t0 of the same group.

*d* = *P* < 0.01 *vs* t0 of the same group.

SURV patients showed a progressive increase in both sperm concentration and total sperm count during the whole follow-up period (*P <* 0.050, T0 *vs* T2). No changes were observed in terms of progressive motility and sperm morphology. On the other hand, RT patients displayed a significant decrease of all these parameters after one year from RT compared to both baseline and the corresponding time point of the SURV group. However, a progressive trend towards the recovery of sperm parameters at baseline was observed after two years of follow-up.

The analysis of sperm aneuploidies during the follow-up period is reported in Table 3. No difference was observed between SURV and RT at baseline. One year on from RT, aneuploidies of sex chromosomes in RT patients underwent an almost 9-fold increase compared to baseline (respectively 5.59 ± 7.85% T1 *vs* 0.57 ± 0.09% T0; *P <* 0.05) whilst aneuploidies detected for autosomes showed a parallel 2.6 fold increase (1.80 ± 0.54% T1 *vs* 0.69 ± 0.15% T0; *P <* 0.05). Although these values returned to nearly baseline levels after 2 years post-orchiectomy, as observed for the other sperm parameters, the rate of total aneuploidies in RT patients was still significantly higher compared to both baseline and the corresponding time point of the SURV group (1.7 ± 1.23% T2 RT *vs* 1.47 ± 0.44% T0 RT and 1.37 ± 0.74 T2 SURV; all *P* values < 0.05). No significant changes of sperm aneuploidies were observed in SURV throughout the follow-up period.

**Table 3:** Analysis of sperm aneuploidies during the follow-up period in patients undergoing surveillance only (SURV, *N* = 61) or radio-therapy (RT, *N* = 131) as post-orchiectomy treatment

	Sex chromosomes aneuploidies (%)		Autosomes aneuploidies (%)		Total aneuploidies (%)	
	SURV	RT	SURV	RT	SURV	RT
T0	0.75 ± 0.82	0.57 ± 0.094	0.82 ± 0.34	0.69 ± 0.15	1.53 ± 1.21	1.47 ± 0.44
T1	0.83 ± 0.45	5.59 ± 7.85 ***b.d***	0.71 ± 0.26	1.80 ± 0.54 ***a.c***	1.55 ± 0.74	7.41 ± 8.61 ***b.c***
T2	0.75 ± 0.42	0.98 ± 1.12 ***c***	0.56 ± 0.21	0.72 ± 0.12	1.37 ± 0.74	1.7 ± 1.23 ***a.c***

Abbreviations: T0: basal, T1: 1 year follow-up, T2: 2 years follow-up significance:

*a* = *P* < 0.05 *vs* corresponding time point of SURV.

*b* = *P* < 0.01 *vs* corresponding time point of SURV.

*c* = *P* < 0.05 *vs* t0 of the same group.

*d* = *P* < 0.01 *vs* t0 of the same group.

### Analysis of hormonal parameters at follow-up

The time course of hormonal parameters and testicular volume of both SURV and RT during the two years of follow-up after orchiectomy and RT are reported in Table 4. For both RT and SURV groups the mean levels of total testosterone (T) never fell below the threshold of hypogonadism (10.4 nmol/L), in RT patients this serum parameter was significantly reduced at 2 years of follow-up (*P <* 0.050.05 *vs* T0 RT and *vs* T2 SURV). Concerning the serum levels of gonadotrophins, SURV patients showed a progressive growth in FSH levels that increased significantly at 2 years of follow-up (13.2 ± 7.1 IU/L 2 years *vs* 8.3 ± 4.6 IU/L baseline, *P <* 0.01). In SURV patients, values of FSH≤8 IU/L at baseline were not prognostic for the development of FSH > 8 IU/L at 2 years of follow-up (OR = 0.28; 95% CI: 0.03–2.5). No gross alteration was reported for LH levels in these patients. Contrarily, RT patients showed a significant and persistent increase of FSH at both one and two years of follow-up (17.7 ± 4.6 IU/L RT T2 *vs* 14.2 ± 8.6 IU/L T1 and 7.1 ± 3.8 IU/L T0; all *P* values < 0.05). In particular in RT patients, values of FSH ≤ 8 IU/L at baseline were protective for the maintenance of FSH ≤ 8 IU/L at 2 years of follow-up (OR = 2.95; 95% CI: 0.93–9.37). Also LH levels in RT patients progressively increased across the two years of follow-up from 6.9 ± 3.6 IU/L to 9.2 ± 5.7 IU/L (respectively *P <* 0.050.05 *vs* T2 SURV and *P <* 0.050.05*vs* RT T0). In particular, at 2 years of follow-up the prevalence of patients showing subclinical hypogonadism (T ≥ 10.4 nmol/L and LH ≥ 8 IU/L [12]) was significantly higher in RT patients compared to SURV (respectively 59/131 *vs* 13/61; *P <* 0.001). Compared to simple surveillance, exposure to radiotherapy was associated with a 3.03 fold risk of developing subclinical hypogonadism (95% CI: 1.50–6,11).

**Table 4:** Hormonal parameters and testicular volume at follow-up in patients undergoing surveillance only (SURV, *N* = 61) or radio-therapy (RT, *N* = 131) as post-orchiectomy treatment

	Total testosterone (nmol/L)		FSH (IU/L)		LH (IU/L)		Testicular volume (mL)	
	SURV	RT	SURV	RT	SURV	RT	SURV	RT
T0	15.8 ± 4.8	16.1 ± 4.6	8.3 ± 4.6	7.1 ± 3.8	5.6 ± 2.8	6.9 ± 3.6	16.5 ± 6.3	16.3 ± 6.6
T1	16.2 ± 4.4	14.5 ± 5.4	10.1 ± 5.2	14.2 ± 8.6 ***a.d***	5.9 ± 3.3	8.3 ± 3.7 ***a***	18.4 ± 5.4 ***c.d***	14.5 ± 5.1 ***a.c.d***
T2	17.4 ± 5.6	12.9 ± 4.4 ***a.c***	13.2 ± 7.1 ***c***	17.7 ± 4.6 ***a***	6.9 ± 4.0 ***c***	9.2 ± 5.7 ***a.c***	20.3 ± 6.1 ***b***	13.4 ± 4.9 ***a.c***

Abbreviations: T0: baseline, T1: 1 year follow-up, T2: 2 years follow-up, FSH: follicle stimulating hormone, LH: luteinizing hormone, nmol/L: nanomoles per liter, mL: milliliter significance:

*a* = *P* < 0.05 *vs* corresponding time point of SURV.

*b* = *P* < 0.01 *vs* corresponding time point of SURV.

*c* = *P* < 0.05 *vs* t0 of the same group.

*d* = *P* < 0.05 *vs* t2 of the same group.

Finally, RT patients showed a progressive reduction of the volume of the remaining testis during the whole follow-up period (16.3 ± 6.6 mL T0 *vs* 14.5 ± 5.1 mL T1 and 13.4 ± 4.9 mL T2; all *P* values < 0.05). An opposite trend was observed in SURV patients, showing a progressive increase across the follow-up period (16.5 ± 6.3 mL T0 *vs* 18.4 ± 5.4 mL T1 *P <* 0.050.05 and 20.3 ± 6.1 mL T2 *P <* 0.01).

### Analysis of the calcium-phosphorous metabolism parameters at follow-up

The time course of calcium-phosphorous parameters during the two years of follow-up are reported in Table 5. Both SURV and RT patients showed a progressive decrease of serum levels of 25(OH) Vit D during the follow-up period. However, in RT patients the extent of this reduction at two years from orchiectomy was more severe (55.5 ± 25,3 nmol/L SURV vs 34.2 ± 23.1 nmol/L RT respectively, *P <* 0.001). Also the prevalence of vitamin D insufficiency (serum 25(OH) Vit D <50 nmol/L [13]) at two-years of follow-up in RT patients was significantly increased compared to SURV (29/61 SURV vs 110/131 RT, *P <* 0.001). In particular, compared to simple surveillance, exposure to radiotherapy was associated with a 5.78 fold risk of developing vitamin D insufficiency (95% CI: 2.91–11.48). On the other hand, parathormone (PTH) showed an opposite trend being significantly increased in both SURV and RT at 2 years of follow-up compared to baseline. However, serum levels of PTH in RT patients at this time point were higher than those observed in SURV patients (66.7 ± 36,1 ng/L SURV vs 72.9 ± 26.8 ng/L RT, *P <* 0.001). Significant variations of serum 1,25(OH)_2_ Vit D, Calcium and Phosphorous were not observed between the two groups and along with the follow-up.

**Table 5:** Parameters of calcium-phosphorous metabolism at follow-up in patients undergoing surveillance only (SURV, *N* = 61) or radio-therapy (RT, *N* = 131) as post-orchiectomy treatment

	25(OH) Vit D (nmol/L) [*Vit D insufficiency n*; %]		PTH (ng/L)		1.25-(OH)_2_ Vit D (nmol/L)		Calcium (nmol/L)		Phosphorous (nmol/L)	
	SURV	RT	SURV	RT	SURV	RT	SURV	RT	SURV	RT
T0	72 ± 32.5 [*17; 27.9%*]	68.7 ± 21.8 [*55; 28.6%*]	38.9 ± 16.4	41.3 ± 21.1	93.8 ± 37.6	92.5 ± 31.2	2.37 ± 0.09	2.30 ± 0.11	1.09 ± 0.14	1.21 ± 0.18
T1	66.7 ± 31.2 [*20; 32.8%*]	49.3 ± 34.3 ***a,c*** [115; 59.9%^***^]	41.8 ± 18.3	52.4 ± 30.7 ***a,c***	94.6 ± 25.5	93.6 ± 32.1	2.39 ± 0.12	2.32 ± 0.15	1.11 ± 0.09	1.14 ± 0.20
T2	55.5 ± 25.3 ***c*** [29; 47.6%^*^]	34.2 ± 23.1 ***b,c*** [110; 83.8%^***^]	66.7 ± 36.1 ***c***	72.9 ± 26.8 ***b,d***	95.8 ± 33.3	95.4 ± 28.4	2.34 ± 0.14	2.44 ± 0.13	1.03 ± 0.13	0.97 ± 0.16

Abbreviations: T0: basal, T1: 1 year follow-up, T2: 2 years follow-up, 25(OH) Vit D: 25-hydroxy Vitamin D, PTH: parathormone, ng/dL: nanograms per deciliter, 1,25(OH)_2_ Vit D: 1,25-di-hydroxy Vitamin D. significance:

*a* = *P* < 0.05 *vs* corresponding time point of SURV.

*b* = *P* < 0.01 *vs* corresponding time point of SURV.

*c* = *P* < 0.05 *vs* t0 of the same group.

*d* = *P* < 0.01 *vs* t0 of the same group.

^*^ = *P* < 0.05 *vs* t0 of the same group at chi-square test.

^***^= *P* < 0.001 *vs* t0 of the same group at chi-square test.

## DISCUSSION

Adverse effects associated with post-orchiectomy treatments for TGCTs represent a major matter of concern because of their potentially deleterious effects on spermatogenesis, sperm aneuploidies and gonadal hormonal function [14]. In this study we provide evidence that post-orchiectomy RT severely impairs spermatogenesis within one year from treatment, an effect that displays some degree of persistance after two years of follow-up. This alteration is accompanied by a reduction of testis volume and a compensatory increase of FSH levels. Most importantly, RT exposes orchiectomized patients to a three-fold greater risk of developing subclinical hypogonadism and a six-fold greater risk of developing vitamin D insufficiency within two years post treatment.

The fact that testis cell populations are highly sensitive to radiation is not new. By inducing the accumulation of DNA double-strand breaks, radiation triggers short-term or mid-term apoptotic events as soon as the cell enters the proliferation or division phase. This event represents the main cytotoxic mechanism by which radiotherapy negatively alters rapidly dividing cells, such as spermatogenetic cells [15]. However, despite the high turnover rate, type A spermatogonia display a different behavior. In fact, after single radiation doses of 0.2–4 Gy, they progressively achieve their nadir levels over 21 weeks [16, 17]. To explain this effect, it has been hypothesized that only a sub-population of the non-cycling A stem spermatogonial cells expresses the lethal damage when they are recruited into cycle. Accordingly, the differentiation of spermatogonia into spermatocytes is reduced during this time, indicating a different damage or signaling from somatic cells [18]. This effect appears reversible since type A spermatogonia undergo repopulation after 21 weeks from formation, with a concomitant ability to differentiate into spermatocytes and at later stages, indicating that self-renewal exceeds cell loss. The rate of sperm count recovery largely depends on the dose of radiation and generally takes up to 24 months to return to pre-irradiation levels after a single dose of 1 Gy and longer after higher doses of irradiation [18]. It should be noted that radiotherapy uses fractioned irradiation, showing even more harmful effects than acute irradiation for the same dose [18] Our data are largely in agreement with this cell model, since RT patients nearly halve their total sperm count at 1 year of follow-up and partially restore spermatogenesis after 2 years from treatment. However, this recovery is not invaluable for the overall quality of sperm cells as highlighted by the significant worsening of cell motility and morphology one year on from RT. These results are in line with an increased rate of sperm aneuploidies detected equally at one year from the treatment. Importantly, the amount of total aneuploidies detected in RT patients at two years of follow up remained significantly increased compared to the SURV counterpart, confirming the few available studies on this topic [19–22]. This evidence gains particular importance during the possible seek of fertility in testis cancer survivors. Accordingly, the counseling of testicular cancer patients should provide information on the risk of possible chromosomal anomalies transmitted to the newborn and the precaution to delay conception likely up to more than 12 months after radiotherapy as previously suggested [21]

An aspect that deserves particular attention is the endocrine function of the testis, beyond the mere production of testosterone [11]. Male hypogonadism is associated with increased prevalence of metabolic disorders, increased risk of mortality for cardiovascular complications and bone alteration [23, 24]. However, Leydig cells are actually involved in several endocrine patterns beyond testosterone production, such as Vit D 25-hydroxylation [11]. An even milder impairment of Leydig cell function, characterized by unvaried serum testosterone levels but increased LH - a clinical condition named subclinical hypogonadism - has been associated with a higher risk of hypovitaminosis D, secondary hyperparathyroidism and osteoporosis [25–31]. Here we report that at two years from orchiectomy, the prevalence of subclinical hypogonadism in RT patients is nearly doubled compared to the control population of SURV. This associates with an almost doubled prevalence of vitamin D insufficiency in RT patients and in significantly increased levels of serum PTH at the same time point of the follow-up. Similar data have also been reported recently, for example, in a study on 36 TGCTs patients who had been RT-treated, Eberhard *et al.* documented an increased prevalence of subclinical hypogonadism persisting even after three years from treatment [10]. On the other hand, Schepisi *et al.* found reduced vitamin D levels after ten years of follow-up in a very small group of RT-treated TGCTs survivors [10, 32]. Of note, the long-term effects observed in the present study are likely to be ascribed to the sole impairment of the testis function. Kidney failure can be excluded by the lack of alteration of serum 1,25(OH)2 Vit D [33]. Compared to SURV, RT patients are thus expected to be exposed to a significantly higher risk of osteoporosis and bone fracture which is major risk factor for increased mortality and reduced quality of life, as reported for TGCTs patients receiving cisplatin therapy [34, 35]. In this regard, the main limitation of this study is the lack of a direct measurement of bone status in our study group. Nonetheless, the evidence highlights the importance of an adequate long-term monitoring of metabolic functions and bone status in TGCT survivors. Indeed, although the efficacy of RT as therapeutic tool in seminoma treatment is beyond the scope of this study, it should be noted that the tumor relapse rate in SURV patients is nearly four times higher than that observed in RT treated patients [36]. Thus all these elements should be critically explained and discussed during oncological counseling in order to correctly address the risk/benefit ratio of RT in TGCT patients [37].

**Figure 1:**
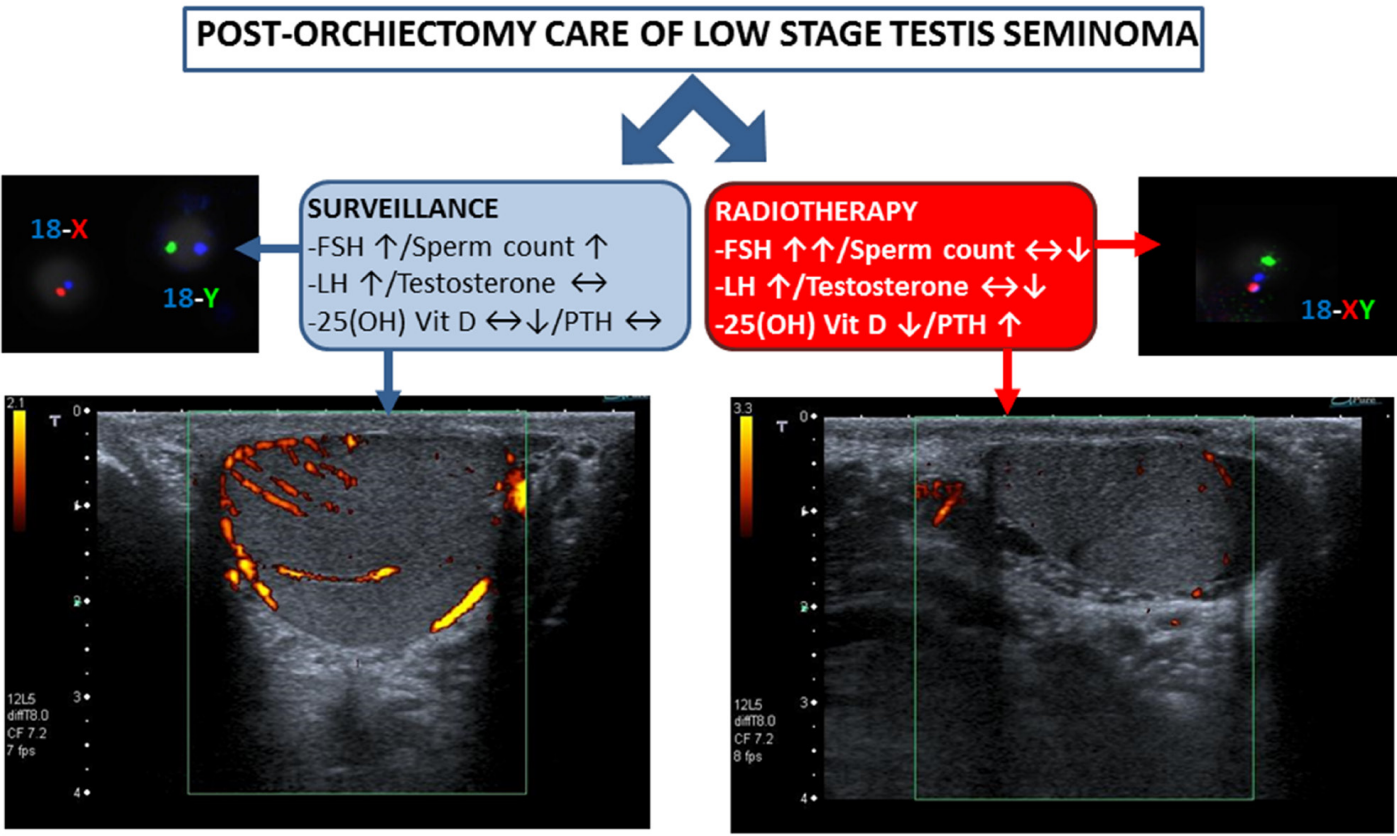
Effects of surveillance and radiotherapy in post-orchiectomy TGCTs are reported Surveillance associates with increased levels of FSH with compensatory stimulation of spermatogenesis and increased testis volume with maintenance of normal vascular pattern. After radiotherapy, persistent increase of FSH accompanies early impairment of spermatogenesis and increased rate of sperm aneuploidies. Surveillance also associates with modest increase LH levels with no variation of testosterone production and possible alteration of calcium-phosphorous metabolism. Radiotherapy exposes to higher risk of subclinical hypogonadism and severe alteration of calcium-phosphorous metabolism with increased risk of osteoporosis related to hypovitaminosis-D.

In conclusion, the effects of surveillance and radiotherapy in post-orchiectomy TGCTs can be summarized as in Figure 1. (1) Surveillance associates with increased levels of FSH with compensatory stimulation of spermatogenesis and increased testis volume. This is not observed after radiotherapy where stronger and persistent increase of FSH accompanies early impairment of spermatogenesis followed by some degree of recovery. (2) Surveillance associates with a modest increase in LH levels with no variation of testosterone production and slight, if any, alteration of calcium-phosphorous metabolism. On the other hand, radiotherapy exposes the patient to a higher risk of subclinical hypogonadism (featured by stronger increase in LH levels even with slight or absent decrease of testosterone) and, most importantly, with severe alteration of calcium-phosphorous metabolism. Secondary effects ascribable to RT are increased and persistent rate of sperm aneuploidies and increased risk of osteoporosis related to hypovitaminosis D. Other studies with a longer follow-up period are necessary to disclose whether subclinical hypogonadism persists in association with other signs or symptoms of premature male frailty.

## MATERIALS AND METHODS

### Subjects

This retrospective study enrolled 192 subjects, all of whom had stage I testis seminoma and were admitted to the Unit of Andrology and Reproductive Medicine (University of Padova, Italy) for gamete cryopreservation between February 2011 and February 2015. All patients completed the study. According to current clinical guidelines on adjuvant treatment in TGCT survivors, treatment options in stage I seminoma patients include RT, SURV, and adjuvant chemotherapy [5]. Patients were divided into two groups based on the post-orchiectomy management: 131 were treated with pelvic RT (RT group) whereas 61 received no other treatment (SURV group). All patients receiving RT had a conformal radiotherapy of para-aortic lymph nodes with 6–15 MV photon beams using CT-assisted three-dimensional treatment planning. RT treatment was administered within one month from orchiectomy according to good clinical practice [5]. A mean radiation dose of 24 Gy was delivered in 12–15 fractions within 16–25 days.

Clinical data of patients were evaluated at baseline (T0), considered as the beginning of RT treatment and the subsequent follow-up after 12 and 24 months (T1 and T2, respectively) for sperm parameters, sex hormones, sperm aneuploidies, testicular volume and serum parameters of calcium-phosphorous metabolism. In this retrospective analysis only patients with the complete panel of clinical data were considered. The exact timing of follow-up visits were 11.8 ± 2.3 months and 23.2 ± 4.1 months. Patients with known causes of infertility such Klinefelter syndrome, Y chromosome microdelections and history of varicocele, orchitis or cryptorchidism were excluded from the study. Written informed consent was obtained from all patients, and the study protocol was approved by the ethical committee for clinical trials of the University Hospital of Padova.

### Semen sample collection and processing

All semen samples were evaluated according to World Health Organization (WHO) guidelines for semen analysis 2010 [38] by a single trained technician (M.M.). Semen samples were obtained by masturbation after 2–5 days of sexual abstinence. After liquefaction at room temperature, semen volume, pH, sperm concentration, total sperm count (TSC), viability, motility, and normal morphology were determined. In particular, sperm morphology was evaluated according to Kruger’s criteria, adapted to WHO 2010 threshold values [38]. Semen samples were then washed three times with sterile phosphate-buffered saline, and the pellet was used for the subsequent analyses.

### Hormone assay

Blood samples were collected in the fasting state between 08:00 and 10:00 h. Serum FSH, LH and T were evaluated by commercial electrochemiluminescence immunoassay methods (Elecsys 2010; Roche Diagnostics, Mannheim, Germany) as reported elsewhere [37, 39]. For all parameters, the intra- and inter-assay coefficients of variation were <8 and 10% respectively. parathyroid hormone (PTH) serum levels were determined with a direct, two-site, sandwich type chemiluminescent immunoassay (LIAISON N-TACT PTH, DiaSorin Inc. Stillwater, MN). 25(OH) Vit D was determined with direct, competitive chemiluminescent immunoassay (LIAISON 25 OH Vitamin D TOTAL Assay, DiaSorin Inc). 1,25(OH)2D was quantified with IDS 1,25-dihydroxyvitamin D RIA Kit (ImmunoDiagnosticSystem, UK), based on purification of 1,25(OH)_2_ Vit D by immunoextraction and quantitation with ^125^I RIA

### Ultrasound (US) scanning

To evaluate testicular size, morphology and normal tissue echo pattern, all subjects were evaluated with ultrasonography. All US examinations were made using a high resolution machine (Aplio XV Toshiba, Tokio, Japan) equipped with a 7–13 MHz multifrequency linear probe by the same experienced physician (P.P.). Testis volume was estimated according to the prolate ellipsoid formula (Length × Width × Height × 0.52 [40]) currently used as the default procedure equipped in echographic software. Intra-observer variability for testicular volume was estimated to be less than 10% as reported in previous studies of our group [35, 37].

### FISH analysis for sperm aneuploidy

The study of sperm aneuploidy was performed by multicolor FISH, as reported elsewhere [41]. DNA hybridization was performed using a human satellite probe-specific mix for autosomes 13, 18, 21 and sex chromosomes X, Y (Kreatech Diagnostics, Amsterdam, The Netherlands). Probes were directly labeled with a specific fluorochrome for each chromosome. The protocol of FISH staining, including sperm nucleus decondensation, DNA denaturation of sperm, incubation with probes, post-hybridization washing and nuclear counter-staining with 6-diamino-2-phenylindole (DAPI) was performed according to the manufacturer’s indications. Slides were finally evaluated with the use of a fluorescence microscope (Nikon Eclipse E600) equipped with a triple band-pass filter set. Single spots were evaluated as reported elsewhere [33]. For each patient, at least 2,500 cells were scored.

### Statistical analysis

Statistical analysis of the data was conducted with SPSS 21.0 for Windows (SPSS, Chicago, IL). The results are expressed as means ± standard deviation (SD). The Kolmogorov–Smirnov test was used to check for normality of distribution. Variables not showing normal distribution were log transformed. Comparison between characteristics of SURV and RT patients at baseline and follow-up were compared by using unpaired Student’s *t* tests with Bonferroni- Holm correction for multiple comparisons. Repeated-measures ANOVA was performed to test for differences in serum and semen parameters during the study at three time points. The Levene’s test was used to test the homogeneity of variance among groups. Where the homogeneity of variance assumption was violated, Welch test was performed and the respective *p* value was reported. The proportion of patients with subclinical hypogonadism and vitamin D insufficiency at the end of the study was compared with Chi-square test. *P* values <0.05 were considered as statistically significant.
